# Effects of the Uncertainty of Interpersonal Communications on Behavioral Responses of the Participants in an Immersive Virtual Reality Experience: A Usability Study

**DOI:** 10.3390/s23042148

**Published:** 2023-02-14

**Authors:** Shirin Hajahmadi, Gustavo Marfia

**Affiliations:** 1 Department of Computer Science and Engineering, University of Bologna, 40126 Bologna, Italy; 2 Department of the Arts, University of Bologna, 40126 Bologna, Italy

**Keywords:** human-computer interaction, virtual reality, serious game, interpersonal communications, uncertainty, social well-being, behavioral responses

## Abstract

Two common difficulties which people face in their daily lives are managing effective communication with others and dealing with what makes them feel uncertain. Past research highlights that the result of not being able to handle these difficulties influences people’s performance in the task at hand substantially, especially in the context of a social environment such as a workplace. Perceived uncertainty of information is a key influential factor in this regard, with effects on the quality of the information transfer between sender and receiver. Uncertainty of information can be induced into the communication system in three ways: when there is any kind of information deficit that makes the target message unclear for the receiver, when there are some requested changes that could not be predicted by the receiver, and when the content of the message is so interconnected and complex that it limits understanding. Since uncertainty is an inseparable feature of our lives, studying the effects that different levels of it have on individuals and how individuals nevertheless accomplish the tasks of daily living is of high importance. Modern technologies such as immersive virtual reality (VR) have been successful in providing effective platforms to support human behavioral and social well-being studies. In this paper, we suggest the design, development, and evaluation of an immersive VR serious game platform to study behavioral responses to the uncertain features of interpersonal communications. In addition, we report the result of a within-subject user study with 17 participants aged between 20 and 35 and their behavioral responses to two levels of uncertainty with subjective and objective measures. The results convey that the application successfully and meaningfully measured some behavioral responses related to exposure to different levels of uncertainty and overall, the participants were satisfied with the experience.

## 1. Introduction

Most of us would agree that uncertainty, in many circumstances, is not something we like to experience. For example, we do not wish to be uncertain about our ability to pay bills at the end of each month, our work and educational prospects, and our health [1]. Some studies in neuroscience also support this claim by providing evidence that the human brain is hardwired to interpret uncertainty as a danger and respond to it with fear and stress [2,3]. A human brain under uncertainty tends to overestimate and dramatize danger [4], jump to conclusions [5], and underestimate its ability to handle it [6,7,8].

Following this approach to uncertainty, the goal has been to reduce it [9,10]. For example, people are encouraged to reduce the uncertainty of loss of income in old age or of possible unemployment with saving money, paying taxes, and buying insurance policies [1]. In education, traditionally, uncertainty is often seen as a threat and removed by exposing students to clearly defined problems, following predefined methods of solving them, to reach expected outcomes [11]. The reality is that we live in an uncertain and complex world [1]. Despite our best efforts, things do not always go as planned, and unexpected events may happen. Hence, one should strive to accept uncertainty, performing tasks aware of its existence instead of amplifying its fear with the risk of arguing with life rather than living it. The recent experience with COVID-19 supports such an idea [12]. This is why many educators have recently sought the best ways to provide a structured and supportive learning environment to prepare young students to respond productively to the challenges originating from dealing with uncertainty [13,14]. As described by Beghetto [15], novel learning environments should structurally offer uncertainty, engaging students with it, teaching them how to sit with its difficulty, how to explore, how to generate and evaluate new possibilities, and, most importantly, take action based on them [16]. In this way, uncertainty may act as a catalyst for creative answers rather than an unbeatable barrier. This approach motivates the idea of designing and implementing platforms to support the study of the behavioral responses that the uncertainty may trigger [12,17,18].

The broad concept of uncertainty is, in fact, closely connected with that of information which, in turn, is at the core of interpersonal communications [19,20]. Interpersonal communication concerns the study of social interaction between people and tries to understand how verbal and written dialogues, as well as nonverbal actions, are used to achieve communication goals [21]. Studies show individuals facing different levels of uncertainty have different behavioral responses, from negative to positive [22,23,24]. The ways a human being may deal with an uncertain situation may differ based on individual differences [25], culture [26], and the level of expertise [27]. Hillen et al presented a conceptualization of an individual’s experience of uncertainty based on a categorization of potential responses [28]. In such a model, ambiguities or/and complexities generate(s) stimuli to the information system. Uncertainties appear when individuals perceive (consciously become aware of) their existence. Cognitive, emotional, and behavioral responses then follow such a perception.

Virtual Reality (VR) systems may act as feasible platforms to assist in understanding behavioral responses to the uncertainty of interpersonal communications, as they may provide 3D spaces involving the same kind of navigational and communication challenges experienced in the real world [29]. With VR, it is possible to create structured environments where the ability of people to cope with challenges can be observed, behavioral data gathered, eventual achievements and feedback engineered, and strategies for skill improvement applied in a top-down fashion [30,31,32,33]. In VR, people can express their ideas, feel in control, and accomplish tasks and communicate with others [34,35,36,37]. This raises the potential to enjoy and engage in activities in the digital space and then apply them to the real world to improve one’s social well-being [38]. In addition, creating such an experience in the context of a serious game can support situated cognition by contextualizing a player’s experience in an engaging and realistic environment [39]. In addition, it can benefit from those game design techniques that support the idea that uncertainty could potentially maintain a user’s attention and engagement, providing the motivation to continue even in challenging moments [1].

Considering this domain, we propose the design and development of an immersive virtual reality experience whose scope is to support the investigation of how people manage uncertainty while performing tasks in a workplace scenario. This experience, implemented as a serious game, aims at simulating a workplace scenario, a social environment where success in managing effective interpersonal communication appears very important [40,41,42,43,44].

With this work, we aim to contribute to the research community by providing answers to the research questions below:

- RQ1: How do the participants rate their experience with different tasks in terms of perceived uncertainty?
- RQ2: How do different degrees of uncertainty affect users’ behavior and performance in this immersive virtual workplace scenario?
- RQ3: How are the users’ subjective responses to uncertainty related to the objective responses?
- RQ4: How does the user evaluate the quality of his/her experience?

This paper is structured as follows. Section 2 discusses related work. Section 3 describes the interaction techniques, environment, and task design process of this immersive VR serious game. Section 4 describes the result of a usability study that evaluates the user experience of the proposed VR system as well as reports some behavioral responses. Section 5 discusses the main findings of the experiment. Section 6 concludes the paper and discusses future opportunities for research.

## 2. Related Work

In this section, we present and discuss the works that fall closest to our contribution. A good body of research has focused on the study of “Navigational uncertainty” and its effect on the user’s spatial navigation performance and behavior [45,46,47,48]. In this area of research, uncertainty has been mostly introduced into the system by creating a perception of disorientation [49] and curing conflict [50] for the user, resulting in an increase in his/her information-seeking behavior. In their recent review, Keller et al. [51] proposed that collecting and analyzing continuous navigational data obtained from the participants in virtual reality experiences that create navigational uncertainty can potentially provide important insight into their information-seeking behavior. For example, in this research [46], the authors focused on the “Looking around behavior” as a common type of information-seeking behavior of participants when experiencing navigational uncertainty. They recorded continuously the heading direction and tried to find its relation to navigational success measures. From this body of literature, we could conclude the potential and importance of the data that could be captured from VR experiences to provide insights into the behavioral responses of people, especially in the study of the effects of a variable, such as uncertainty, on behavior.

Another area in which the study of uncertainty has received a lot of attention is gaming. As Costikyan et al. [1] claim, games could improve by purposefully applying the concept of uncertainty in their designs. Uncertainty could act as a catalyst to hold users’ attention and interest; mastering it may help pursue a game’s goal in an efficient and non-threatening way [52]. In addition, Costikyan et al. [1] support these claims by citing the sociologist Roger Callios [53] “Play is… uncertain activity. Doubt must remain until the end, and hinges upon the denouement… every game of skill, by definition, involves the risk for the player of missing his stroke and the threat of defeat, without which the game would no longer be pleasing. The game is no longer pleasing to one who, because he is too well trained or skillful, wins effortlessly and infallibly”.

In the following, we review some examples of games that exploit uncertainty in their design and present a comparison of their features in Table 1:

- Gone Home [54] is a first-person exploration game designed to put players in unknown situations, engaging them to stay and accomplish some tasks, such as uncovering the narration by non-linear progression through searching the space. This game puts a player in the shoes of a young woman who returns home and finds that her family is absent. As Veale et al. [55] also discussed, Gone Home is a video game that uses effective storytelling to create empathy and a sense of responsibility in users by placing them within a recent historical moment. In this way, it exposes the user to the positive and negative elements of the past and encourages him/her to stay in the game and reflect on these elements [56]. While not strong on interactivity, the game through a careful visual, spatial, and audio design of the environment leads its users to explore the house along a twisting, uncertain path and find out what happened to the woman’s family through an analysis of imperfect clues from the memorabilia, journals, and other items left around the various rooms. During the experience, there are notes, voices, and letters from or to her family that motivate and guide her in the exploration. These items of cues can be kept in the inventory and reviewed whenever desired [54]. Considering an interest in the study of navigational behaviors of users, Bonnie Ruberg [57] argues that with a deeper analysis of the interactive elements of the game, the player path is linear instead of meandering despite what it seems the game encourages players to do. The path is already set and the locked, or hidden doors prevent the user to have access to some areas unless they trigger an event or find an object that unlocks this barrier in a predefined order.
- Don’t Starve [58] is a survival game that places the user in the role of a scientist who finds himself in a strange and unfamiliar world. The goal is to collect and effectively use survival tools. An uncertain scenario amounts to the interaction with the frogs in the game, as this creates ambiguity, as it is unclear whether they are hostile. For example, they can represent food, but different outcomes may result from eating them. The game successfully engages the user to accept this ambiguity till effectively able to develop higher-level strategies to interact with them. Farah et al. [59] studied the multiplayer expansion of the game to track cooperative features and teamwork behavioral markers.
- Wenge xu et al. [60] developed a motion-based survival game, GestureFit, that involves the user in a fight with a monster. They induced uncertainty in the system through three uncertain game elements: false attacks (creating the perception that there would be a chance that the system is tricking the player to waste a defense move by defending against a false attack), misses (creating the perception that there would be a chance that the actual hit will be interpreted as a miss), and critical hits (creating the perception that there would be a chance that an attack would be a critical attack and produce more damage than a normal one). In this way, they created two different levels of uncertainty, one with inducing these uncertain elements into the game and the other without. After, they conducted a study to measure the effects of levels of uncertainty (certain and uncertain), the display type (VR and LD), and age (young adults and middle-aged adults) on the game experience, performance, and exertion level. Their results showed that for the kind of game they designed, virtual reality could improve game performance. In addition, they found that the uncertain elements that they applied in their design might not help enhance the overall game experience, but could help increase the user’s exertion.
- RelicVE [61] is a virtual reality (VR) game that gives the user a similar role to an archaeologist and engages him in an exploration process of an archaeological discovery experience. It exploits uncertainty in the design of their exploration process by placing the user in a situation where s/he does not know the shape and features of the target artifact and only can discover it by gradually and strategically using available tools and physical movements. In this way, when the user hits specific triggers, a new part of the information about the artifact will be uncovered. They also managed the complexity of the game by the complexity of the shape and volume of the artifact. They integrated VR interaction techniques in the design of their virtual system to create an experience close to the real-world experience of archaeologists and in this way increased the immersion and physical activity of the user during the experience. In addition, they used a timer and a health bar to add the element of time pressure to the experience. To evaluate the experience, the authors also conducted a usability study that found the experience to be innovative as it can improve players’ learning and motivation by adding the elements of uncertainty into the design.

To the best of our knowledge, no previous work took full advantage of the available technologies, such as virtual reality, to induce structured uncertainty and investigate the influence of uncertainty levels on human behavior with a focus on interpersonal communications. Our study tries to take this step from within the design and development of such an application by applying some of the design techniques inspired by the previous games in this area and virtual reality techniques that improve the user experience and the study of behavior.

## 3. Experimental Setting

**Figure 1:**
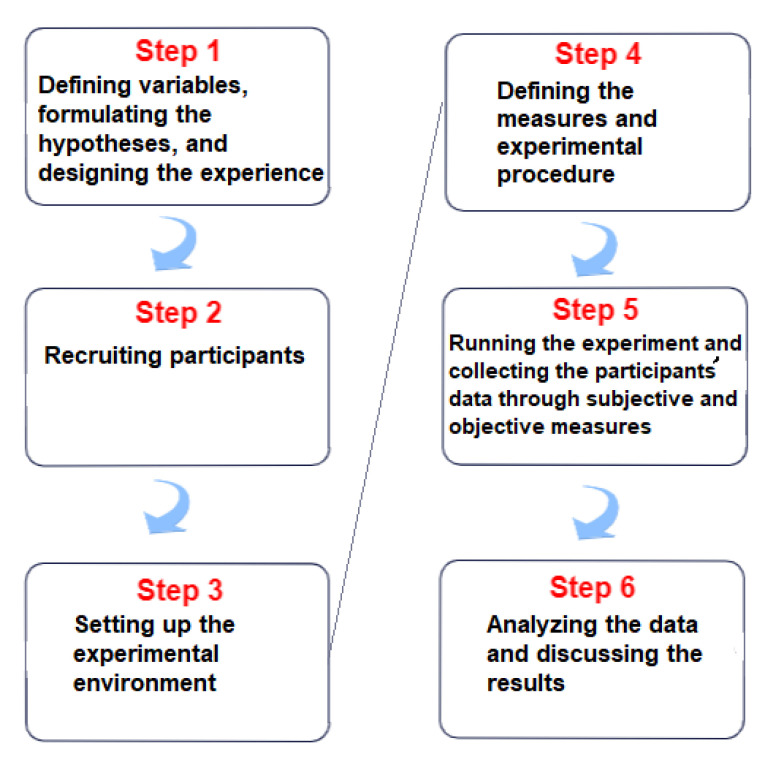
The stages of the experimental design.

In this section, we describe the experiment we conducted to study the effects of different levels of uncertainty on behavioral responses, performance, and quality of the experience of the participants resorting to objective and subjective measures. Figure 1 visualizes the stages of this experimental design.

### 3.1. Participants

We recruited 17 participants (3 female, 14 male, age: 20–35, M = 25.05, SD = 1.75) who are affiliated with our university to take part in our study. Detailed demographic information appears in Table 2. Before starting our experiment, we asked them to rate (1 = never, 5 = every day) any previous experience with virtual reality using a head-mounted display (M = 2.88, SD = 1.36) and their level (1 = low, 5 = high) of English proficiency (M = 3.47, SD = 1.01).

### 3.2. Materials

We now proceed to present the design and implementation choices of the virtual environment.

#### 3.2.1. Setup

In our experiment, participants navigated in a virtual office via an HTC Vive Pro HMD (refresh rate: 90 Hz, resolution: 1440 × 1600 pixels, FoV 110°) connected to a workstation (Intel(R) Core(TM) i7-6850K CPU @ 3.60 GHz, 3.60 GHz). The environment was developed using Unity 3D version 2019.4.35 f1. Unity 3D is a game engine developed by the Unity Technologies (San Francisco, CA, USA). It is a very famous platform that has been used by game developers across the world. The data analysis was performed using R version 4.2.2 and RStudio version 2022.07.2+576. R is a programming language and software environment for statistical computing and graphics, developed by the R Development Core Team and maintained by the R Foundation (Vienna, Austria). RStudio is an integrated development environment (IDE) for the R programming language, developed by RStudio, Inc. (Boston, MA, USA).

#### 3.2.2. Experience Design

The experience was designed as a role-playing serious game where a user, in the role of a new employee, is exposed to two different levels of uncertainty in the context of interpersonal communication in a workplace scenario. To this aim, the story plot that develops within the experience takes inspiration from Amelia Bedelia, the protagonist and title character of the children’s book series authored by Peggy Parish [62]. Amelia Bedelia is a housekeeper who takes her instructions literally because her boss could not be present in the house on the first day of her work. The instructions include lexical ambiguity coming from each sentence. Despite such ambiguity, Amelia stays positive and expresses her excitement to do her job well and make her boss happy, but she repeatedly misunderstands the guidance. Inspired by this story line, our application implements:

- An absence of guidance when a user is following and executing given instructions. This design limits the access to sources of information, asking the user to focus and rely on the already provided knowledge or information that may come after from sources such as panels and phone calls;
- Specific means of communicating instructions, which may be textual with the use of panels (and sometimes verbal, e.g., through phone calls) as a result of the absence of guidance;
- Specific instruction communication patterns in the form of sequences of sentences, such as what appears in step-by-step construction manuals. At the same time, it enables an experiment designer to purposely reduce the amount of available information and change the complexity of the sentences to control the amount of ambiguity and complexity in the system;
- The possibility of applying lexical ambiguity, as another potential source of confusion;
- A friendly environment supporting understanding and empathy in interpersonal communications.

Please see the Table 3 for a comparison between these features in the proposed platform and Amelia Bedelia story.

**Figure 2:**
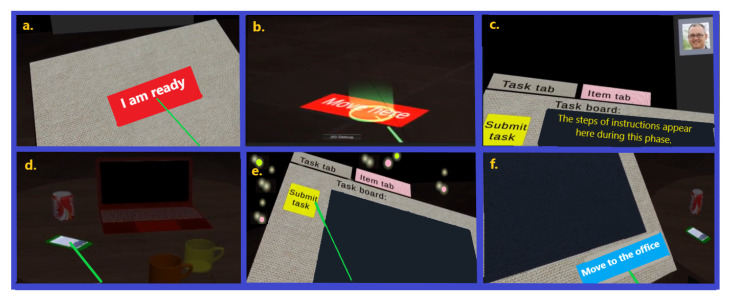
Screenshots showing some steps of the familiarization phase, from left to right: (**a**) selecting the “I am ready” button by the user; (**b**) teleporting to a destination; (**c**) reading instructions of the task from the panel on the user’s left hand; (**d**) removing an interactive object; (**e**) submitting the current task; (**f**) selecting the “Move to the office” button to move there.

The application includes two phases: the “Familiarization” phase and the “Main” phase. The goal of the “familiarization” phase is to remove any uncertainty arising from unfamiliarity with VR interfaces and the related context. For this purpose, it provides information and a step-by-step tutorial with feedback to familiarize the user with the context and allow the user to feel confident with the interactions that will then be executed. The user in this phase will get to know the boss, his/her role, the space s/he will be working in, the means of communication, and the way s/he can accomplish the tasks indicated by the boss using the available interfaces. At the end of this phase and when the system confirms the user has successfully executed all steps, s/he will reach the virtual office by pressing the “Move to the office” button from the panel on the left hand (see Figure 2 for some screenshots taken from the familiarization phase).

The main phase starts with the user finding himself/herself inside a virtual office in front of a door. After 10 s, a phone starts ringing, and s/he should answer. The boss is on the phone, welcoming and asking the player to follow some instructions, explaining three options that will be available during the experience: submitting the task, suppressing it, and requesting help using the buttons on the panel. The boss also says that if something important comes up he will call again. By pressing the “I am ready” button on the panel, a description of the first task appears. The user can now teleport to move within the office environment, removing objects based on the instructions. The removed objects then become visible in the “Item” tab of the panel. The user can cancel a previous removal by pressing the close button near each image. The user will be asked to complete a second task either by pressing the “Submit task” button or the “I do not do this task” button. After removing a specific number of objects, in the middle of the second task the phone will ring. The boss warns the user that it may be necessary to cancel previous removals to follow a new set of instructions. Task 2 finishes either by pressing the “Submit task” button or the “I will not do this task” button. The user can also decide to exit the game by pressing the “Exit the game” button.

**Figure 3:**
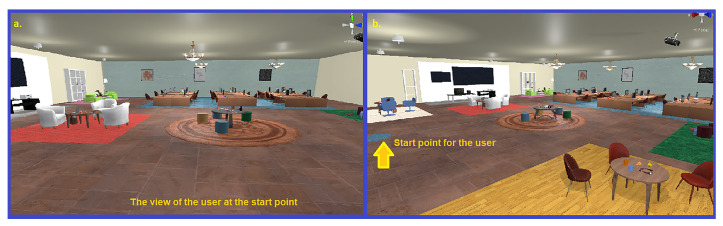
Screenshots showing two views of the office: (**a**) the view of the office from the perspective of the user at the beginning; (**b**) Another view of the office.

**Figure 4:**
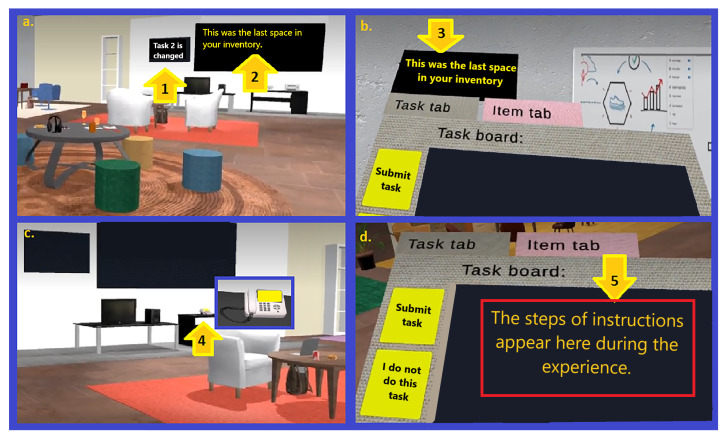
Screenshots showing five sources of information for the user during the experience: (**a**) Arrow 1 points to a small blackboard that displays the name of the current task. Arrow 2 points to a big blackboard that communicates the current status of the tasks during the experience; (**b**) Arrow 3 points to a small blackboard attached to the panel that is a closeup of the big one; (**c**) Arrow 4 points to a phone that blinks and rings when the boss calls; (**d**) Arrow 5 points to a task board that shows the instructions for each task.

**Figure 5:**
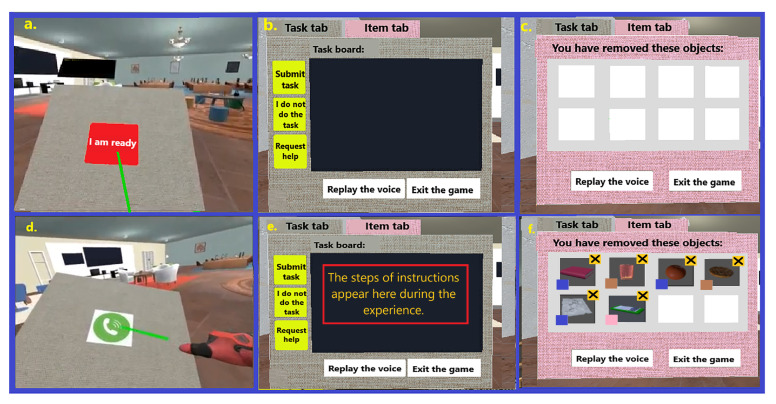
Screenshots showing different parts of the panel for interactions: (**a**,**d**) selecting the “I am ready” and “Answering the phone” buttons by the user; (**b**,**e**) visualization of the task tab; (**c**,**f**) visualization of the item tab.

**Figure 6:**
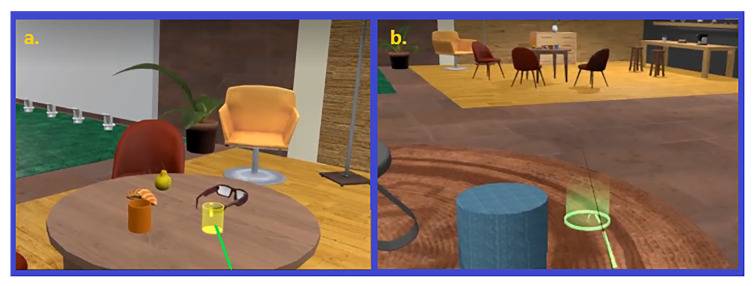
Screenshots showing the participant when: (**a**) removing interactive objects; (**b**) using the teleportation to move in the environment.

The virtual office is hence furnished with interactive and non-interactive objects as well as two dynamic blackboards as two sources of information (See Figure 3 for some screenshots showing the virtual office environment). As described in Figure 4, there are five possible sources of information in the experience: 1. a small blackboard displays the name of the current task; 2. a big blackboard communicates the current status; 3. a small blackboard attached to the panel is a closeup of the big one; 4. a phone that blinks and rings when the boss calls; and 5. a task board showing the instructions for each task. The different parts of the panel are shown in Figure 5. An example of teleporting and removing interactive objects may be viewed in Figure 6. In addition, to increase the immersion, during the main phase an ambient sound is played, simulating the sounds coming from nearby offices to help reduce the confounding effects of noises coming from the real world.

The tasks amount to sequences of instructions to search and remove objects expressed in written form or verbally at different moments in the experience. The tasks include two levels of uncertainty, as inspired by the definition of Hillen et al. [28]. As explained before, uncertainty appears in terms of ambiguity, probability, and complexity. Ambiguities result from incomplete guides and instructions. Probable situations appear as unexpected task changes, and complexity as a change in the number of causal factors in the instructions. Following this guide, we proposed these two tasks to represent two levels of uncertainty: 

Task 1 (or base task): This includes a simple and clear set of instructions. For each step, the number, place, and color of objects that should be removed are clearly expressed.

Instructions for Task 1:

- Step 1: Go to the blue rug area. Remove the red glasses and green smartphone from the desk.
- Step 2: Go to the blue rug area. Remove the apple and orange from the tables.
- Step 3: Go to the brown rug area. Remove the blue cup from the table.
- Step 4: Go to the red rug area. Remove the green wallet and the sandwich from the table.
- Step 5: Go to the white rug area. Remove the pink book from the table.

**Figure 7:**
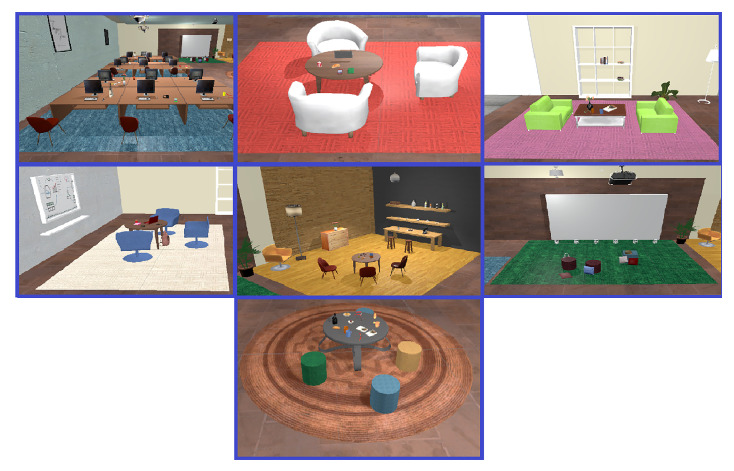
Screenshots from seven different areas for the placements of objects characterized by the color of their associated rugs.

Task 2 (or with an intermediate level of uncertainty): This is a task whose degree of uncertainty includes more complex instructions when compared to Task 1. This Task comprises lexical ambiguity in the instruction, instructions with missing information, and the possibility of instruction changes on the fly (See Figure 7 for the placements of objects in seven different areas of the environment with their associated rugs).

Instructions for Task 2:

- Step 1: Go to the brown rug area. Remove any food not positioned on a plate from the table.
- Step 2: Remove the glasses from the brown rug area.
- Step 3: If you find some mugs in the pink rug area, remove the orange one.
- Step 4: Go to the green rug area. Remove the pillows that are closest to the hat.
- Step 5: If you find calculators on the table and a bag under the table in the white rug area, remove the calculators.

Instructions for Task 2 (after change):

- Step 1: Go to the brown rug area. Remove any food on plates on the table.
- Step 2: Remove the glasses (if you find them there) from the brown rug area.
- Step 3: If you find any books in the blue rug area, remove any pencils near them.
- Step 4: Go to the red rug area. If a bag lies under the table, do not remove the smartphone.
- Step 5: If you see any mugs on the table in the yellow rug area, remove them.

### 3.3. Methods

#### 3.3.1. Procedure

**Figure 8:**
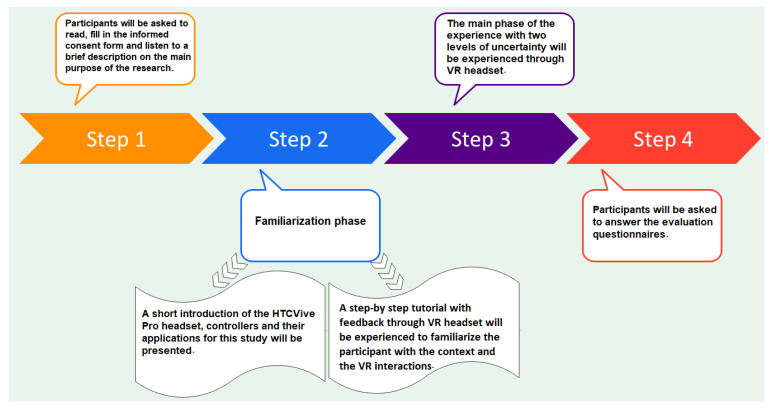
The steps that the participant experiences.

After the participants read the consent form and provided their informed consent, we briefly explained that the experience would develop in a virtual office and that they would be asked to perform some tasks there. Then, a short introduction of the HTC Vive Pro headset, controllers, sensors, and their applications for this study was provided. Then, the users started with the familiarization phase and were guided to the main phase of the experiment. Afterward, the users answered demographic and evaluation questions that will be analyzed later. Figure 8 provides a diagram showing the steps that the participant experiences.

#### 3.3.2. Measures

In this section, we describe the objective and subjective measures used to test our hypotheses.

Objective measures

The application records behavioral responses that could be inferred from the HTC Vive controllers and the headset log data. The following variables were measured:

- Variables related to the time:
  − Time to submit Task 1;
  − Time to submit Task 2;
  − Response time to new messages in Task 1;
  − Response time to new messages in Task 2.
- Variables related to the position: Position of the user in each moment.

Subjective measures

We utilized multiple questionnaires to evaluate participants’ subjective experience with the application as detailed below.

- The Demographic questionnaire: This questionnaire asked participants about their nationality, sex, age, and education level.
- Level of English proficiency questionnaire: A five-point Likert scale was used to rate the level of English proficiency of participants (See Table 4).
- Previous Experience with immersive VR: A five-point Likert scale was used to rate the previous experience with immersive VR of participants (See Table 4).
- Perceived uncertainty questionnaire: In this questionnaire, using a five-point Likert scale, participants were asked to rate their level of perceived uncertainty for each task after the experiment (See Table 4).
- System Usability Scale (SUS) questionnaire: This questionnaire [64] consists of 10 items and utilizes a scale of 1 (Strongly disagree) to 5 (Strongly agree) to provide a “quick and dirty” reliable tool for measuring usability.
- Slater–Usoh–Steed presence questionnaire (SUS): This questionnaire [65] consists of five items and utilizes a scale of one to seven to assess participants’ sense of being there in a virtual office.
- The immersive experience questionnaire (IEQ): This questionnaire [66] comprises 31 items and utilizes a scale of 1 (Not at all) to 7 (A lot) to measure the subjective experience of being immersed while playing a virtual serious game.
- Motion sickness questionnaire (MSAQ): This questionnaire [67] comprises 16 items, utilizes a scale of 1 to 9, and is a valid instrument for the assessment of motion sickness.
- Intolerance of Uncertainty Scale (IUS): This questionnaire [68] consists of 27 items and utilizes a scale of 1 (Not at all characteristic of me) to 5 (Entirely characteristic of me) that assesses emotional, cognitive, and behavioral reactions to ambiguous situations, implications of being uncertain, and attempts to control the future.

## 4. Results

**Figure 9:**
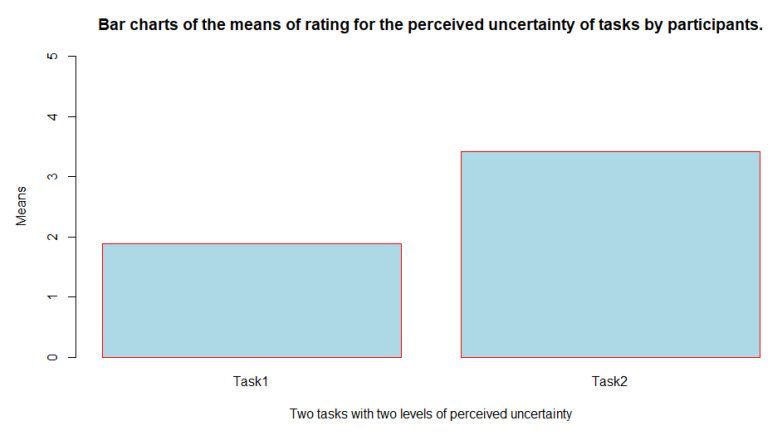
A comparison between the means of ratings obtained from the participants for their perceived uncertainty of Task 1 (M = 1.88, SD = 1.11) and Task 2 (M = 3.41, SD = 1.00); Range of answers = 1–5.

In this section, we present the objective and subjective results of our experiment concerning our research questions:

We compared the ratings that the participants gave to the perceived uncertainty of two tasks with the Wilcoxon signed-rank test. The result found a significant difference between them (v = 0, *p* = 0.0003553 < 0.05), suggesting that overall the participants rated Task 2 with higher perceived uncertainty than Task 1 (See also Figure 9 for a visual comparison of the ratings).

**Figure 10:**
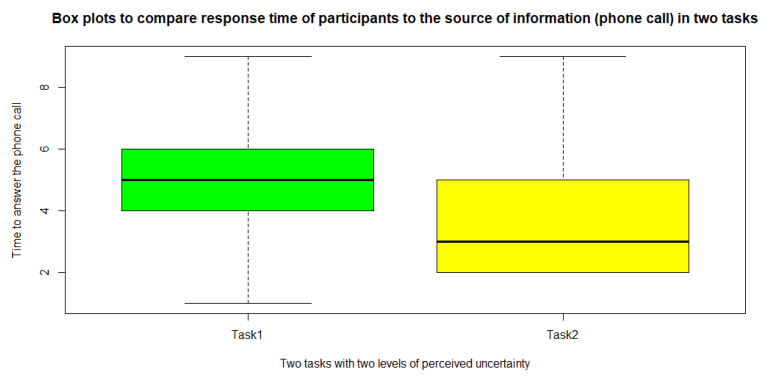
A comparison between the response time of the user to the source of information (the phone call) during Task 1 and Task 2, measured in [s].

To find the effects of different degrees of induced uncertainty on the user’s behavior, we first confirmed the normality of the data with the Shapiro–Wilk test at the 5% level. Then, we conducted the Paired *t*-test. The results did not yield a significant difference between the response time in the two tasks (t(16) = 1.44, *p* = 0.084 > 0.05). However, the box plot in Figure 10 visually shows a lower response time to pick up the phone in Task 2 when compared to Task 1.

**Figure 11:**
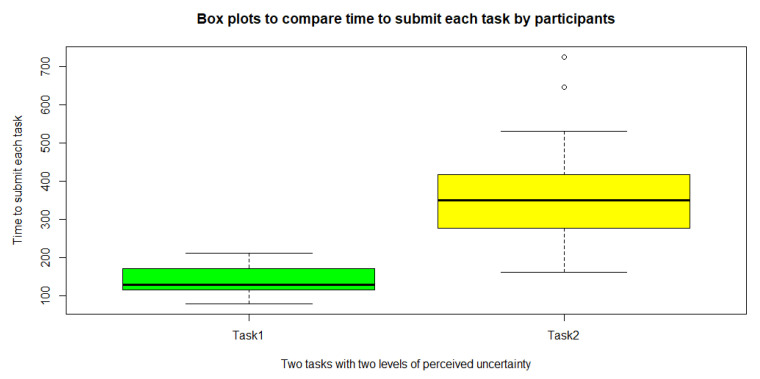
A comparison between the task time completion of the user for Task 1 and Task 2, measured in [s]. In these data, two completion time values identified as outliers which are shown in white circles in the figure.

Since the normality of the data was rejected by the Shapiro–Wilk test at the 5% level, using the Wilcoxon signed-rank test (v = 0, *p* = 0.00001526 < 0.05), we found a significant difference between the task completion time for Task 1 and Task 2 (See also Figure 11 to see a visual comparison between the amounts).

**Figure 12:**
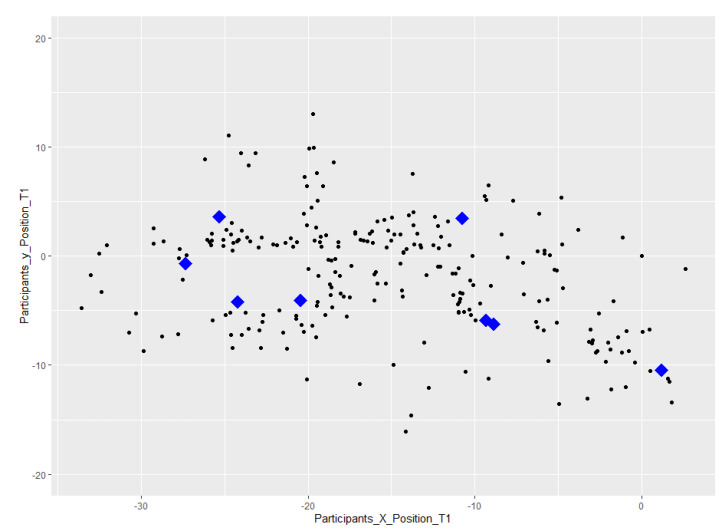
A visualization of participants’ change of position in Task 1; the 8 task targets are shown in blue.

**Figure 13:**
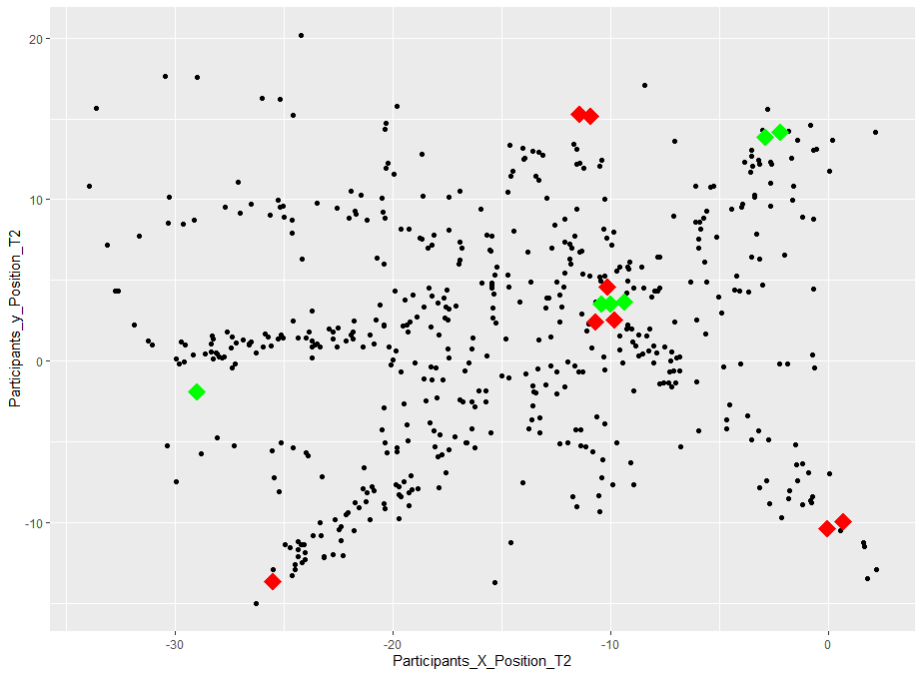
A visualization of participants’ change of position in Task 2; the task targets related to before the change of the task are shown in red; the task targets related to after the change of the task are shown in green.

To report the differences in the change of position in Task 1 in comparison to Task 2, Figure 12 and Figure 13 present a visual comparison of participants’ change of position.

We used Pearson’s r-test to measure the strength and direction of the possible linear relationship between the scores on system usability, immersion, presence, motion sickness, and intolerance of uncertainty questionnaires and the recorded time to answer the second call (i.e., response time to the source of information in Task 2). We also used this test for finding the possible relationships between the scores of these questionnaires and the time spent on the second task. See Table 5 for the results of these tests.

**Figure 14:**
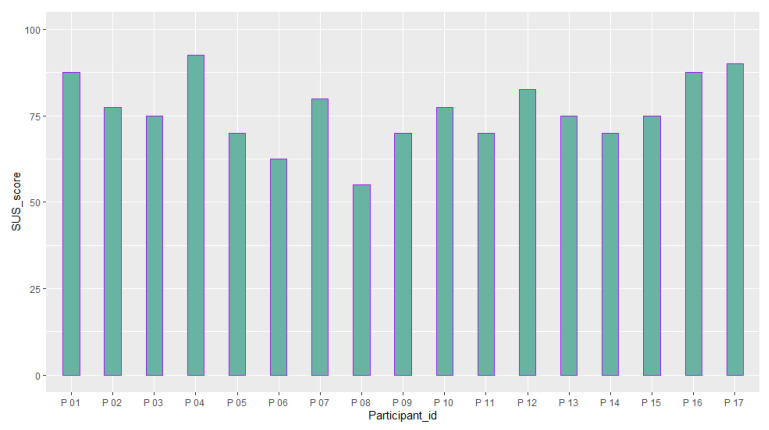
A comparison between the scores obtained from the participants from the System Usability Scale (SUS) questionnaire (M = 76.32, SD = 9.89).

**Figure 15:**
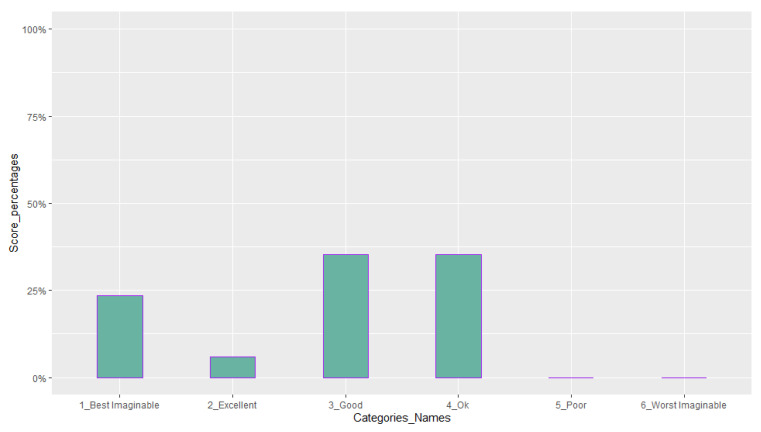
Percentages of ratings for each category from the System Usability Scale (SUS) questionnaire, categories from left to right are: best imaginable (score > 84.1); excellent (72.6 < score < 84.0); good (62.7 < score < 72.5); ok (51.7 < score < 62.6); poor (26 < score < 51.6); worst imaginable (score < 25).

**Figure 16:**
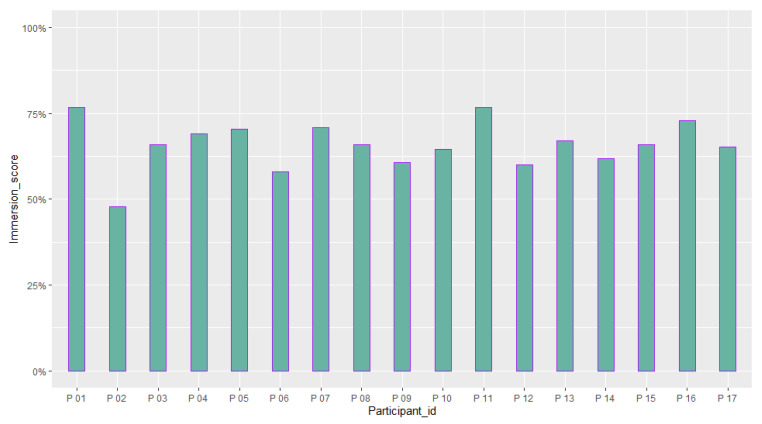
A comparison between the scores obtained from the participants from the Immersive Experience questionnaire (IEQ) (M = 65.84 (%), SD = 7.13 (%)).

**Figure 17:**
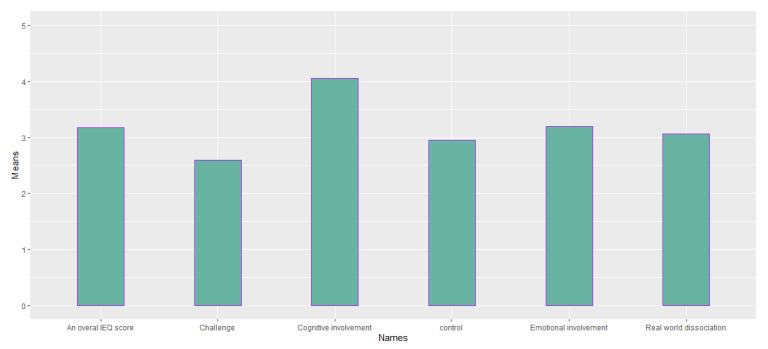
Range of (1: Not at all; 5: A lot) for a comparison between the means of scores for the components of the Immersive Experience questionnaire (IEQ) scale, from left to right: overall IEQ score (M = 3.17); challenge (M = 2.59); cognitive involvement (M = 4.06); control (M = 2.95); emotional involvement (M = 3.20); and real-world dissociation (M = 3.059).

**Figure 18:**
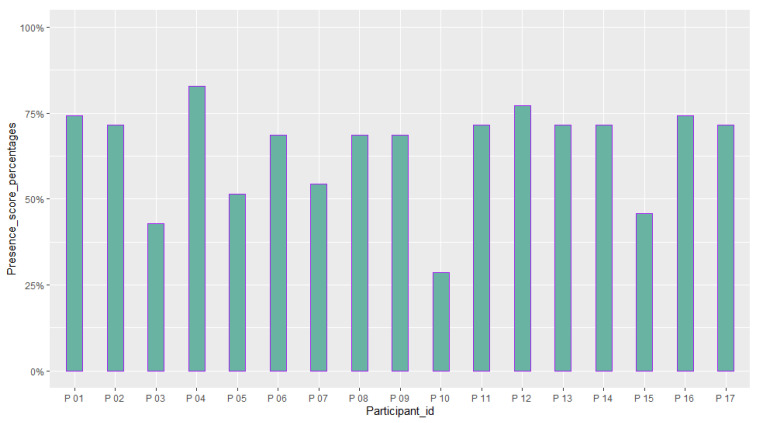
A comparison between the scores obtained from the participants from the Slater–Usoh–Steed presence (SUS) questionnaire (M = 64.37 (%), SD = 14.50 (%)).

**Figure 19:**
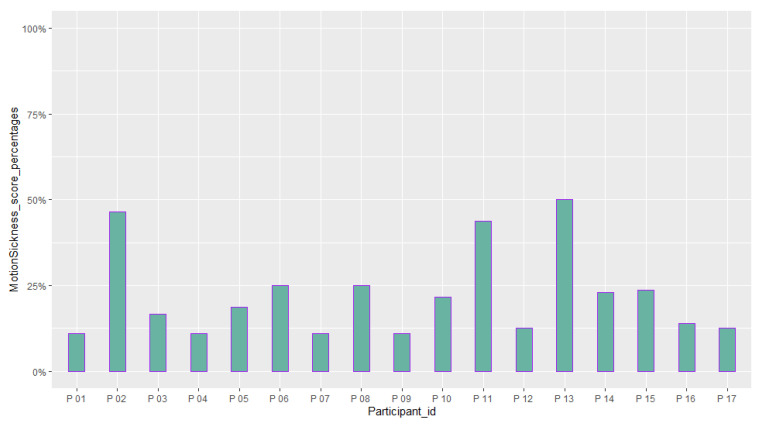
A comparison between the scores obtained from the participants from the Motion Sickness questionnaire (MASQ) (M = 22.18 (%), SD=12.84 (%)).

**Figure 20:**
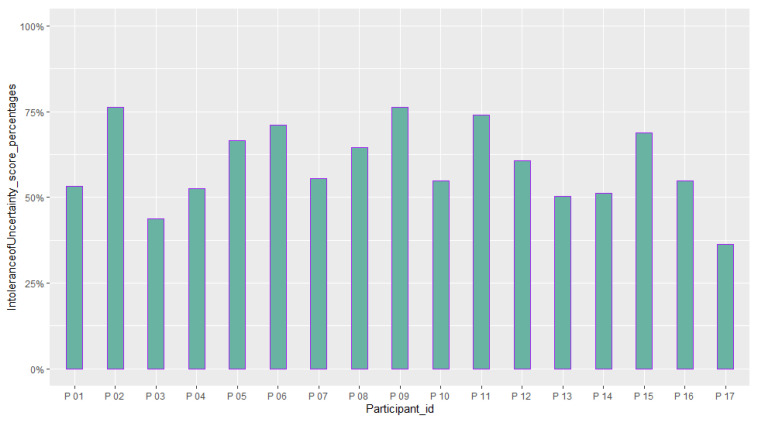
A comparison between the scores obtained from the participants from the Intolerance of Uncertainty Scale (IUS) (M = 59.48 (%), SD=11.63 (%)).

Table 6 reports the mean and standard deviation of scores obtained from the questionnaires about the quality of the participants’ experience and their intolerance to uncertainty. Figure 14, Figure 15, Figure 16, Figure 17, Figure 18, Figure 19 and Figure 20 present a visual comparison of the data obtained.

## 5. Discussion

In this section, we present and discuss the main findings of the experiment in more detail.

The main purpose of this study was to suggest the design and implementation of a VR platform that is able to create the experience of uncertainty of interpersonal communications on two levels and to record and report human behavioral responses to this exposition. In this paper, we addressed these research questions: 

RQ1: Is there any significant difference between subjective ratings of participants for perceived uncertainty of Task 1 and Task 2?

Our findings from a comparison of the post-experiment ratings of the participants to the perceived uncertainty of two tasks indicate the potential of the proposed design to successfully produce at least two levels of uncertainty in the experience of the system. 

RQ2: How do different degrees of induced uncertainty affect the users’ behavior and performance in this immersive virtual workplace scenario?

- RQ2-1: Does the response time of the user to reach the source of information (the phone call) differ in the two tasks?
- RQ2-2: Does the task completion time for the user for each task differ?
- RQ2-3: How does the change of participants’ position in Task 1 differ from Task 2?

In this paper, we targeted the study of the differences between behavioral responses to the experience of two levels of uncertainty. In particular, we focused on studying the real-time records of the time and position of the participants. 

Related to time, we were interested in two variables:

1. Response time: In particular, we were interested in the participant’s response time to new information coming from a highly influential source that was directly associated with the boss, a phone call. Our expectation was that by increasing the degree of induced uncertainty, the participant would show a different response time, but we did not find a significant difference in this comparison with the applied statistical test.
2. Task completion time: In particular, we were interested in the time that the participants persist in accomplishing each task as an objective measure of the user’s tolerance to the change of uncertainty in the system. For this reason, we did not consider the correctness or wrongness of following the instructions, and the user was free to end tasks at any moment without experiencing any time pressure or encouraging or discouraging feedback. Related to this setting, by increasing the level of uncertainty of the task, our expectation was that the participant spends a different amount of time accomplishing the task. The results of the study were aligned with this expectation by reporting a significantly higher completion time for Task 2.

Related to position: The positions of the participants in Task 1 and Task 2 were recorded by the application every 4 s. The distribution of them along two axes of X and Y and in comparison with the targets for each individual task is visualized in Figure 12 and Figure 13. From a visual comparison of the two plots, we can see that in total, participants in Task 2, a task with an increase in uncertainty level, have more changes of position. This finding is aligned with what we were expecting.

In sum, our experiment suggests that adding uncertainty to a task will harm task performance on completion time, but not in response time. 

RQ3: How are the users’ subjective responses to uncertainty related to the objective responses?

Despite our expectations for finding strong relations between the subjective and objective measures, we found a small negative linear correlation between the scores of the system usability scale and time to answer the second call, small positive linear correlations between the presence score and both task completion time for Task 2 and time to answer the second call, and a small positive linear correlation between scores of the motion sickness questionnaire and time to answer the second call. We think with the increase in sample size, we can report stronger correlations between these variables.

RQ4: How does the user evaluate the quality of his/her experience through subjective measures?

Another purpose of the study was to report the results of the participants’ evaluation of their experience with the system. The mean score of our results from the System Usability Scale (SUS) conveys a higher amount than the average SUS score which is 68. This gives an immediate insight into the overall good usability of the system and the need for minor improvements in the design [69]. In addition, based on the adjective rating scale introduced by [70], we also found that nearly 70 % of the participants’ ratings of the usability of the system fit into “Best imaginable”, “Excellent”, and “Good” categories (See Figure 15). For the Immersive Experience questionnaire (IEQ), the Slater–Usoh–Steed presence (SUS) questionnaire, and the Motion Sickness questionnaire (MASQ), the average of the scores also falls into an acceptable range representing a good quality of the participants’ experiences (See Figure 16, Figure 17, Figure 18 and Figure 19).

In sum, we can conclude that the system with the help of the designed environment and story plot is able to create a pleasant virtual experience. In addition, with the help of tracing from the HTCVive pro controllers and headset we were able to successfully capture in real time the behavioral responses of the participants related to the time of actions, and user position to our variable of interest.

## 6. Conclusions and Future Works

In this paper, we investigated the effects of uncertainty level in a virtual office on participants’ objective and subjective responses through a controlled human-subject study. We designed an experimental scenario inspired by a famous story name Amelia Bedelia written by Peggy Parish [62]. For the design of our system, we first investigated and carefully selected the virtual reality interfaces and environments that supported our research needs. In addition, we were inspired by previous games which applied uncertainty in their designs. The goal was to develop a system that supports a pleasant 3D immersive experience with real-world-like interactions and rich data-collecting techniques. In our usability study, participants were asked to complete two different tasks inside a virtual office where they were also involved in interpersonal communication with their boss on the first day of work. We measured the participants’ objective responses through the log data captured from the tracing of HTCVive pro controllers and headsets as well as assessed their subjective experience through questionnaires. We determined that the two proposed versions of tasks received significantly different ratings from the participants for their perceived uncertainty after the experiment. In addition, our results supported that the time taken to submit different tasks differs significantly. In addition, results from the usability, immersion, presence, and motion sickness questionnaires conveyed that overall, the participants were satisfied with the experience by scoring the usability, presence, and immersion of the experience on average higher than 50% and the motion sickness of the experience less than 30%.

This paper suggested that our proposed VR system can manipulate the levels of uncertainty to study it. In the design of this system, we inspired ourselves from real-life situations. An example workplace scenario could be what happens regularly for one in the role of a manager. S/he may receive multiple unpredictable inputs at once and has to constantly monitor and choose what to do first, stay productive, and successfully monitor time allocations to be able to work with everyone involved [71]. To indicate how effectively our system replicates such real-life happenings under the same conditions, an evaluation of our proposed system against real-life baseline conditions is required. We decided not to consider this system evaluation in this paper because of our limitations in controlling the confounding factors coming from real-world settings that make it hard for us to have a valid measure of the effects of uncertainty. So, we leave it for future work. In addition, we plan to investigate more behavioral responses from the user in a future study and assess the feasibility of this application with a desktop-based version of it. Finally, a larger sample size helps us to report and study more powerful behavioral results of the study.

## Figures and Tables

**Table 1:** Key features of the games reviewed in the related work section.

Name of the Game	Type of the Game	Type of the Experience	Applied Uncertainty Approach	Requested Task
Gone Home	Exploration game; Role-playing game	Video game	Put players in unknown situations; Encourage the user to explore scattered objects along a twisting, uncertain path	To find out what has happened to the character’s sister and family by digging through scattered documents
Don’t Starve	Exploration game; Role-playing game	Video game	Put the user in a strange and unfamiliar world; Put the user in a situation that is uncertain about the outcome of some of his/her choices	To find through trial and error the way that can survive from the threats coming from the environment
GestureFit	Motion-based survival game	Immersive virtual reality	Induce uncertainty in the system through three uncertain gameplay elements (false attacks, misses, and critical hits)	To stay alive and perform gestures to defeat a monster
RelicVE	Exploration game; Role-playing game	Immersive virtual reality	Place the user in a situation where s/he does not know the shape and features of the target artifact from the beginning	To take away earth from the artifact using the available tools and physical movement without damaging it

**Table 2:** Participants’ demographic information.

Sex	Number	Percentage (%)
Female	3	17.65
Male	14	82.35
Age	Number	Percentage (%)
20–30	17	100
30–35	0	0
Nationality	Number	Percentage (%)
Italian	17	100
Others	0	0
Education level	Number	Percentage (%)
High school graduate, diploma or the equivalent	1	5.9
Bachelor’s degree	12	70.59
Master’s degree	3	17.65
Doctorate degree	1	5.9

**Table 3:** A comparison of the elements in the proposed platform with those in the Amelia Bedelia story.

Name of the Element	Description of the Element in Amelia Bedelia Story	Description of the Element in Our platform	Reason for Use
Absence of guidance	The housekeeper is asked to accomplish tasks based on the given instructions while does not have access to anybody to communicate her doubts.	The same situation is true here for the user but also s/he can save in the system the type of problem which is facing (a problem with the interface and/or a problem with the instruction).	This design choice limits the access to the sources of information, asking to focus and rely on the already provided knowledge or may come after, from sources such as panels and phone calls.
Specific means of communicating instruction	Textual on a printed paper	Both textual and verbal that comes from the panels and voice calls	Communicating instructions in both verbal and textual forms would be in favor of cognitive load and managing the attention of the user during the experience [63].
Specific instruction communication patterns and sources of ambiguity	Instructions are presented in sequences of sentences, as appearing in step-by-step construction manuals. These instructions include lexical ambiguity coming from each sentence.	The same is followed here. In addition, the complexity of instructions also changes with increasing the degree of interconnectivity among parts of the sentences. In addition, available tasks and information change with receiving unpredictable phone calls coming from the boss.	This design choice provides the possibility for the experiment designer to purposely reduce the amount of available information and change the complexity of the sentences to control the amount of ambiguity and complexity in the system. In addition, the possibility of applying lexical ambiguity, as another potential source of confusion is provided.
A friendly environment supporting understanding and empathy in interpersonal communications	A nice house with friendly relationships between Amelia and the family	A nice virtual office and the friendly voice of the boss	Experiencing this environment potentially keeps the user’s interest to stay till the end of the experiment and accept the challenges.

**Table 4:** Questionnaires used to rate the level of English proficiency, the previous experience with immersive VR, and the perceived uncertainty of Task 1 and Task 2.

Measuring Item	Question	Range
Level of English proficiency	How do you rate your level of English proficiency?	(1. Poor–5. Very good)
Previous Experience with immersive VR	Have you experienced virtual reality with a head-mounted display in the past?	(1. Never–5. Everyday)
Rating the perceived uncertainty of Tasks 1 and 2	From the definition of Hillen et al. [28], changes in three sources can provide uncertainty in an information system: probability, ambiguity, or/and complexity of information. You can perceive uncertainty if you can become aware of its existence. Based on this definition and your experiment with the virtual office, what score will you give to the perceived uncertainty of Task 1 (the same question for Task 2)?	1–5

**Table 5:** Correlation values between subjective and objective measures.

Questionnaire Name	Time to Answer the Call in Task 2	Task Competition Time for Task 2
The System Usability Scale (SUS)	r = −0.38	r = −0.15
Immersive Experience Questionnaire (IEQ)	r = 0.15	r = 0.4
Slater–Usoh–Steed presence questionnaire (SUS)	r = 0.24	r = 0.29
Motion sickness questionnaire (MASQ)	r = 0.21	r = 0.16
Intolerance of Uncertainty Scale (IUS)	r = −0.09	r = 0.07

**Table 6:** Mean and standard deviation of scores received from participants’ answers to the questionnaires.

Questionnaire Name	Mean	SD	Range
The System Usability Scale (SUS)	76.32	9.89	[1–100]
Immersive Experience Questionnaire (IEQ)	65.84	7.13	[1–100]
Slater–Usoh–Steed presence questionnaire (SUS)	64.37	14.50	[1–100]
Motion sickness questionnaire (MASQ)	22.18	12.48	[1–100]
Intolerance of Uncertainty Scale (IUS)	59.48	11.63	[1–100]

## Data Availability

Available upon request.

## References

[1] Costikyan G. (2013). Uncertainty in Games.

[2] Peters A., McEwen B.S., Friston K. (2017). Uncertainty AND stress: Why it causes diseases and how it is mastered by the brain. Prog. Neurobiol..

[3] Mobbs D., Hagan C.C., Dalgleish T., Silston B., Prévost C. (2015). The ecology of human fear: Survival optimization and the nervous system. Front. Neurosci..

[4] Kessler O., Daase C. (2008). From insecurity to uncertainty: Risk and the paradox of security politics. Alternatives.

[5] Bensi L., Giusberti F. (2007). Trait anxiety and reasoning under uncertainty. Personal. Individ. Differ..

[6] Konstantellou A., Sternheim L., Hale L., Simic M., Eisler I. (2022). The experience of intolerance of uncertainty for parents of young people with a restrictive eating disorder. Eat. Weight Disord.-Stud. Anorex. Bulim. Obes..

[7] Grupe D.W., Nitschke J.B. (2013). Uncertainty and anticipation in anxiety: An integrated neurobiological and psychological perspective. Nat. Rev. Neurosci..

[8] Mason J.W. (1968). A review of psychoendocrine research on the pituitary-adrenal cortical system. Psychosom. Med..

[9] Stirling A. (2010). Keep it complex. Nature.

[10] Stirling A. (2008). Science, precaution, and the politics of technological risk: Converging implications in evolutionary and social scientific perspectives. Ann. N. Y. Acad. Sci..

[11] Lee H.S., Anderson J.R. (2013). Student learning: What has instruction got to do with it?. Annu. Rev. Psychol..

[12] Bacon A.M., Krupić D., Caki N., Corr P.J. (2022). Emotional and Behavioral Responses to COVID-19. Eur. Psychol..

[13] Duch B.J., Groh S.E., Allen D.E. (2001). The Power of Problem-Based Learning: A Practical “How to” for Teaching Undergraduate Courses in Any Discipline.

[14] Pigott J. (2022). Less is more: Education for uncertain times. Glob. Soc. Educ..

[15] Beghetto R.A. (2018). What If?: Building Students’ Problem-Solving Skills Through Complex Challenges.

[16] Beghetto R.A. (2019). Structured uncertainty: How creativity thrives under constraints and uncertainty. Creativity under Duress in Education?.

[17] Piccolo M., Milos G.F., Bluemel S., Schumacher S., Mueller-Pfeiffer C., Fried M., Ernst M., Martin-Soelch C. (2019). Behavioral responses to uncertainty in weight-restored anorexia nervosa–preliminary results. Front. Psychol..

[18] Tanovic E. (2020). Individual Differences in Cognitive, Affective, and Behavioral Responses to Uncertainty. Ph.D. Thesis.

[19] Klir G.J. (2006). Uncertainty and Information: Foundations of Generalized Information Theory.

[20] Berger C.R., Calabrese R.J. (1974). Some explorations in initial interaction and beyond: Toward a developmental theory of interpersonal communication. Hum. Commun. Res..

[21] Berger C.R. (2008). Interpersonal communication. The International Encyclopedia of Communication.

[22] Koerner N., Dugas M.J. (2006). A cognitive model of generalized anxiety disorder: The role of intolerance of uncertainty. Worry and Its Psychological Disorders: Theory, Assessment and Treatment.

[23] Kornilova T.V., Chumakova M.A., Kornilov S.A. (2018). Tolerance and intolerance for uncertainty as predictors of decision making and risk acceptance in gaming strategies of the Iowa gambling task. Psychol. Russ..

[24] Boswell J.F., Thompson-Hollands J., Farchione T.J., Barlow D.H. (2013). Intolerance of uncertainty: A common factor in the treatment of emotional disorders. J. Clin. Psychol..

[25] Andersen S.M., Schwartz A.H. (1992). Intolerance of ambiguity and depression: A cognitive vulnerability factor linked to hopelessness. Soc. Cogn..

[26] Hofstede G. (1980). Culture’s Consequences.

[27] Kuhlmann D.O., Ardichvili A. (2015). Becoming an expert: Developing expertise in an applied discipline. Eur. J. Train. Dev..

[28] Hillen M.A., Gutheil C.M., Strout T.D., Smets E.M., Han P.K. (2017). Tolerance of uncertainty: Conceptual analysis, integrative model, and implications for healthcare. Soc. Sci. Med..

[29] Mandal S. (2013). Brief introduction of virtual reality & its challenges. Int. J. Sci. Eng. Res..

[30] Dede C. (1996). The evolution of distance education: Emerging technologies and distributed learning. Am. J. Distance Educ..

[31] Brookes J., Warburton M., Alghadier M., Mon-Williams M., Mushtaq F. (2020). Studying human behavior with virtual reality: The Unity Experiment Framework. Behav. Res. Methods.

[32] Bohné T., Heine I., Gürerk Ö., Rieger C., Kemmer L., Cao L.Y. (2021). Perception engineering learning with virtual reality. IEEE Trans. Learn. Technol..

[33] Villagrasa S., Fonseca D., Durán J. Teaching case: Applying gamification techniques and virtual reality for learning building engineering 3D arts. Proceedings of the Second International Conference on Technological Ecosystems for Enhancing Multiculturality.

[34] Gugenheimer J., Stemasov E., Frommel J., Rukzio E. Sharevr: Enabling co-located experiences for virtual reality between hmd and non-hmd users. Proceedings of the 2017 CHI Conference on Human Factors in Computing Systems.

[35] Cobbett S., Snelgrove-Clarke E. (2016). Virtual versus face-to-face clinical simulation in relation to student knowledge, anxiety, and self-confidence in maternal-newborn nursing: A randomized controlled trial. Nurse Educ. Today.

[36] Soto J.B., Ocampo D.T., Colon L.B., Oropesa A.V. (2020). Perceptions of ImmerseMe Virtual Reality Platform to Improve English Communicative Skills in Higher Education. Int. J. Interact. Mob. Technol..

[37] Hoffman H., Vu D. (1997). Virtual reality: Teaching tool of the twenty-first century?. Acad. Med. J. Assoc. Am. Med Coll..

[38] Bossard C., Kermarrec G., Buche C., Tisseau J. (2008). Transfer of learning in virtual environments: A new challenge?. Virtual Real..

[39] De Gloria A., Bellotti F., Berta R. (2014). Serious Games for education and training. Int. J. Serious Games.

[40] Moynihan D.P. (2008). Learning under uncertainty: Networks in crisis management. Public Adm. Rev..

[41] Zeidner M., Matthews G., Roberts R.D. (2004). Emotional intelligence in the workplace: A critical review. Appl. Psychol..

[42] Barseli M., Sembiring K., Ifdil I., Fitria L. (2019). The concept of student interpersonal communication. JPPI J. Penelit. Pendidik. Indones..

[43] Marris P. (2003). The Politics of Uncertainty: Attachment in Private and Public Life.

[44] Gudykunst W.D. (1995). Anxiety/Uncertainty Management (AUM) Theory: Current Status. Intercultural Communication Theory.

[45] Stankiewicz B.J., Legge G.E., Mansfield J.S., Schlicht E.J. (2006). Lost in virtual space: Studies in human and ideal spatial navigation. J. Exp. Psychol. Hum. Percept. Perform..

[46] Brunyé T.T., Haga Z.D., Houck L.A., Taylor H.A. (2017). You look lost: Understanding uncertainty and representational flexibility in navigation. Representations in Mind and World.

[47] Brunyé T.T., Gagnon S.A., Gardony A.L., Gopal N., Holmes A., Taylor H.A., Tenbrink T. (2015). Where did it come from, where do you go? Direction sources influence navigation decisions during spatial uncertainty. Q. J. Exp. Psychol..

[48] Herdener N., Wickens C.D., Clegg B.A., Smith C. (2016). Overconfidence in projecting uncertain spatial trajectories. Hum. Factors.

[49] Cheng K., Huttenlocher J., Newcombe N.S. (2013). 25 years of research on the use of geometry in spatial reorientation: A current theoretical perspective. Psychon. Bull. Rev..

[50] Hirsh J.B., Mar R.A., Peterson J.B. (2012). Psychological entropy: A framework for understanding uncertainty-related anxiety. Psychol. Rev..

[51] Keller A.M., Taylor H.A., Brunyé T.T. (2020). Uncertainty promotes information-seeking actions, but what information?. Cogn. Res. Princ. Implic..

[52] Stanley D., Latimer K. (2011). ‘The Ward’: A simulation game for nursing students. Nurse Educ. Pract..

[53] Caillois R. (2001). Man, Play, and Games.

[54] Yap C.M., Kadobayashi Y., Yamaguchi S. (2015). Conceptualizing Player-Side Emergence in Interactive Games: Between Hardcoded Software and the Human Mind in Papers, Please and Gone Home. Int. J. Gaming Comput.-Mediat. Simul. (IJGCMS).

[55] Veale K. (2017). Gone Home, and the power of affective nostalgia. Int. J. Herit. Stud..

[56] Smith L., Campbell G. (2015). The elephant in the room: Heritage, affect, and emotion. A Companion to Heritage Studies.

[57] Ruberg B. (2020). Straight paths through queer walking simulators: Wandering on rails and speedrunning in Gone Home. Games Cult..

[58] (2013). Klei Entertainment. Don’t Starve. Video game. PC, IOS, Android, Xbox, Playstation, Wii U. Klei Entertainment, Canada. https://www.klei.com/games/dont-starve.

[59] Farah Y.A., Dorneich M.C., Gilbert S.B. (2022). Evaluating Team Metrics in Cooperative Video Games. Proceedings of the Human Factors and Ergonomics Society Annual Meeting.

[60] Xu W., Liang H.N., Yu K., Baghaei N. Effect of gameplay uncertainty, display type, and age on virtual reality exergames. Proceedings of the 2021 CHI Conference on Human Factors in Computing Systems.

[61] Liu Y., Lin Y., Shi R., Luo Y., Liang H.-N. Relicvr: A virtual reality game for active exploration of archaeological relics. Proceedings of the Extended Abstracts of the 2021 Annual Symposium on Computer-Human Interaction in Play.

[62] Parish P., Siebel F., Thomas B.S., Canetti Y. (1963). Amelia Bedelia.

[63] Tabbers H., Martens R., van Merriënboer J.J. (2000). Multimedia instructions and cognitive load theory: Split-attention and modality effects. Proceedings of the National Convention of the Association for Educational Communications and Technology.

[64] Brooke J. (1996). SUS-A quick and dirty usability scale. Usability Eval. Ind..

[65] Usoh M., Catena E., Arman S., Slater M. (2000). Using presence questionnaires in reality. Presence.

[66] Rigby J.M., Brumby D.P., Gould S.J., Cox A.L. Development of a questionnaire to measure immersion in video media: The Film IEQ. Proceedings of the 2019 ACM International Conference on Interactive Experiences for TV and Online Video.

[67] Gianaros P.J., Muth E.R., Mordkoff J.T., Levine M.E., Stern R.M. (2001). A questionnaire for the assessment of the multiple dimensions of motion sickness. Aviat. Space Environ. Med..

[68] Buhr K., Dugas M.J. (2002). The intolerance of uncertainty scale: Psychometric properties of the English version. Behav. Res. Ther..

[69] Brooke J. (2013). SUS: A retrospective. J. Usability Stud..

[70] Bangor A., Kortum P., Miller J. (2009). Determining what individual SUS scores mean: Adding an adjective rating scale. J. Usability Stud..

[71] Zika-Viktorsson A., Sundström P., Engwall M. (2006). Project overload: An exploratory study of work and management in multi-project settings. Int. J. Proj. Manag..

